# Applying RE-AIM to examine the impact of an implementation facilitation package to scale up a program for Veterans with chronic obstructive pulmonary disease

**DOI:** 10.1186/s43058-023-00520-5

**Published:** 2023-11-21

**Authors:** Edward C. Portillo, Martha A. Maurer, Jordyn T. Kettner, Sonia D. Bhardwaj, Ziting Zhang, Cassie Sedgwick, Aaron M. Gilson, Jamie A. Stone, Nora Jacobson, Rose Hennessy-Garza, Sarah Will, M. Shawn McFarland, Heather Ourth, Michelle A. Chui

**Affiliations:** 1 School of Pharmacy, University of Wisconsin – Madison, 77 Highland Avenue, Madison, WI 53705 USA; 2 William S. Middleton Veterans Affairs Hospital, 2500 Overlook Terrace, Madison, WI 53705 USA; 3 Institute for Clinical and Translational Research and School of Nursing, University of Wisconsin – Madison, 4240 Health Sciences Learning Center, 750 Highland Avenue, Madison, WI 53705 USA; 4 Zilber School of Public Health, University of Wisconsin – Milwaukee, 1240 N 10th St, Milwaukee, WI 53205 USA; 5 Kansas City Veterans Affairs Medical Center, 4801 Linwood Blvd, Kansas City, MO 64128 USA; 6 Department of Veterans Affairs Pharmacy Benefits Management, Clinical Pharmacy Practice Office, 810 Vermont Avenue NW, Washington, DC 20571 USA

**Keywords:** Chronic obstructive pulmonary disease, RE-AIM, Veterans Healthcare Administration, Implementation facilitation

## Abstract

**Background:**

US Veterans are four times more likely to be diagnosed with chronic obstructive pulmonary disease (COPD) compared to the civilian population with no care model that consistently improves Veteran outcomes when scaled. COPD Coordinated Access to Reduce Exacerbations (CARE) is a care bundle intended to improve the delivery of evidence-based practices to Veterans. To address challenges to scaling this program in the Veterans’ Health Administration (VA), the COPD CARE Academy (Academy), an implementation facilitation package comprised of five implementation strategies was designed and implemented.

**Methods:**

This evaluation utilized a mixed-methods approach to assess the impact of the Academy’s implementation strategies on the RE-AIM framework implementation outcomes and the extent to which they were effective at increasing clinicians’ perceived capability to implement COPD CARE. A survey was administered one week after Academy participation and a semi-structured interview conducted 8 to 12 months later. Descriptive statistics were calculated for quantitative items and thematic analysis was used to analyze open-ended items.

**Results:**

Thirty-six clinicians from 13 VA medical centers (VAMCs) participated in the Academy in 2020 and 2021 and 264 front-line clinicians completed COPD CARE training. Adoption of the Academy was indicated by high rates of Academy session attendance (90%) and high utilization of Academy resources. Clinicians reported the Academy to be acceptable and appropriate as an implementation package and clinicians from 92% of VAMCs reported long-term utilization of Academy resources. Effectiveness of the Academy was represented by clinicians’ significant increases (*p* < 0.05) in their capability to complete ten implementation tasks after Academy participation.

**Conclusions:**

This evaluation found that the use of implementation facilitation paired with additional strategies enhanced the capacity of clinicians to implement COPD CARE. Future assessments are needed to explore post-academy resources that would help VAMCs to strategize localized approaches to overcome barriers.

Contributions to the literature

- Research has shown that COPD interprofessional care programs improve patient functional status and reduce COPD-related hospitalizations. However, there are many barriers to implementing and scaling such services across health systems.
- We found the use of an implementation facilitation package enhanced clinicians’ perceived capacity to successfully implement an interprofessional service titled COPD CARE across medical centers simultaneously.
- These findings contribute to the growing literature documenting the use of implementation facilitation as an effective strategy to promote adoption of interprofessional clinical services.

## Background

Chronic obstructive pulmonary disease (COPD) is an irreversible, progressive, and debilitating respiratory illness characterized by airway inflammation and airflow limitation [1, 2]. It is the fourth leading cause of death and disability [3] and the third leading cause of hospitalizations in the US It is estimated that COPD will become the leading global cause of death by 2033 [4, 5]. The US Veteran population is especially vulnerable to COPD as Veterans are four times more likely to be diagnosed with it, more susceptible to its complications, and have a higher COPD mortality rate compared to the civilian population [6].

Although COPD is not fully reversible, it is treatable when evidence-based approaches to its management such as medication optimization, adherence review, inhaler technique, and symptomatic assessment are used [7]. While these best practices are well-established in the literature and clinical guidelines, it remains a challenge to embed these recommendations into routine primary care delivery models [8, 9]. As a result, only one-third of US patients with COPD receive evidence-based treatment [9]. Barriers to implementing and scaling COPD evidence-based best practices include lack of informatics infrastructure, limited staffing and practitioner engagement, and high workload [9, 10].

COPD care bundles, which combine multiple evidence-based clinical interventions for COPD management into one service [11] have demonstrated positive patient outcomes [12–20] Yet there is a need to better understand the best approaches to promote scale-up of COPD bundles across multiple settings [15].

This evaluation explores whether a virtual implementation package, COPD Coordinated Access to Reduce Exacerbations (CARE) Academy (Academy), built capacity for implementing a COPD care bundle in the Veterans’ Health Administration (VA). The VA is the largest integrated health care system in the United States with 171 VA Medical Centers (VAMCs) with unique processes, cultures, priorities, and geographic barriers that can make scaling best practices difficult.

Initial design of the Academy began in 2018 with the development of a clinical training program that was refined and tested across two VAMCs [16]. The program was found to have a positive impact on clinician confidence and interprofessional collaboration; however, clinicians reported they needed additional guidance and resources to overcome logistical barriers to implementing COPD CARE [17]. Furthermore, a national implementation team, including experts in pharmacy and COPD management, recognized that different implementation strategies were needed to scale COPD CARE more rapidly.

**Fig. 1:**
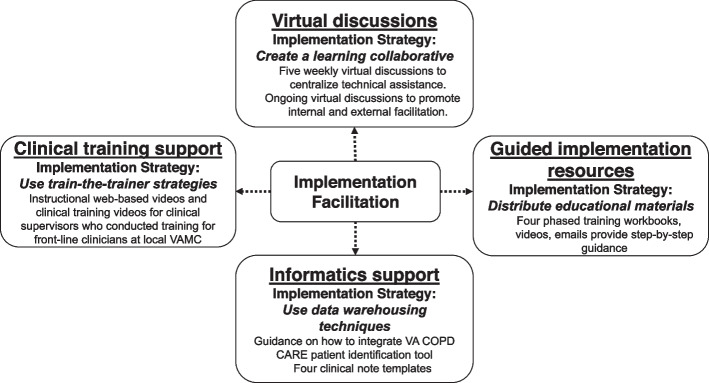
Core components of the Academy

To address these obstacles and promote effective service reach, the national implementation team developed a more comprehensive implementation package over a 12-month period with support from VA experts in Dissemination & Implementation (D&I) science [21]. The Academy is a 5-week virtual training program led by national COPD CARE experts who serve as external facilitators. It is comprised of five discrete implementation strategies [22] including implementation facilitation (IF) (Fig. 1), which is an interactive approach to addressing implementation challenges through forming supportive relationships [23]. The Academy provides facilitation through cohort-based learning by convening clinicians from multiple VAMCs working together to implement the COPD CARE bundle at their respective VAMCs. The Academy trains clinicians and provides them with resources to serve as internal facilitators and support implementation at their VAMCs. The external facilitators are included to promote discussion and shared problem solving. Virtual discussions, guided implementation resources, informatics support, and clinical training support were integrated within the IF model (Fig. 1).

### Evaluation conceptual framework

We applied the RE-AIM conceptual framework [24, 25] to measure the impact of the Academy on building capacity for implementing COPD CARE. RE-AIM emphasizes translating evidence-based interventions into practice and has five domains and associated measures for examining an intervention’s reach (R), effectiveness (E), adoption (A), implementation (I), and maintenance (M). RE-AIM was selected as a guiding framework to assess the Academy impact due to its inclusion of implementation outcomes in addition to effectiveness outcomes [24].

The specific aims of this evaluation were to assess the impact of the Academy on clinicians’ capability to implement COPD CARE (effectiveness) and to assess the reach, adoption, implementation, and maintenance of the Academy as it relates to clinicians’ capability for implementation. Future evaluations are planned to examine more distal outcomes, such as the effect of the Academy on the implementation of COPD CARE as indicated by increased use of best practices and improvements in Veteran care.

## Methods

### Design

This quality improvement evaluation utilized a mixed-methods approach to obtain retrospective feedback from clinicians about their perceptions of the Academy and its impact on their capability to implement COPD CARE. This evaluation was determined not to meet the federal definition of research and qualified for a quality improvement exemption.

### Setting and sample

The Academy was implemented in two cohorts in Fall 2020 and Spring 2021. Cohort one involved five Midwestern VAMCs and Cohort two involved eight VAMCs from the West and East coasts and the Southwest. Two approaches were used to identify the VAMCs: [1] the Academy was promoted through a national VA website known for promoting promising practices to VA leaders [26], and [2] program developers strategically engaged with Clinical Pharmacy Executives across the VA to identify VAMCs with a strong interest in the program. VAMCs that agreed to participate were instructed to complete a pre-implementation workbook, which involved identifying implementation team members, including an *implementation lead*, responsible for guiding the overall process at their site and a *clinician lead* responsible for conducting the front-line clinician training.

### Data sources

Three primary data sources were used for this evaluation: [1] administrative records, [2] a survey, and [3] a semi-structured interview and are described in Table 1.

**Table 1:** Data source, description, and timeframe

Data source	Description	Timeframe
Administrative records	• Information documented by the National COPD CARE team detailing the Academy process and clinical training	Data gathered throughout the evaluation period
Survey	• 34-item Qualtrics survey with five domains: [1] Academy participation, [2] perceptions of the Academy, [3] perceived capability to complete implementation tasks before and after Academy participation, [4] barriers to implementation, and [5] plans for implementation. • Respondents were 16 clinicians who participated in the Academy • Respondents represented 13 VAMCs	Data gathered 1 week after completing the 5-week Academy
Semi-structured interviews	• 38-question Interview to understand clinicians’ experiences applying Academy resources, with three domains: [1] utilization of the Academy and perceptions of Academy content, [2] acceptability of Academy content and delivery, and [3] experiences with COPD CARE implementation. • Eight items used a 7-point Likert scale to rate agreement with statements (i.e., 1 = very strongly agree to 7 = very strongly disagree) • Interviewees were clinicians involved in COPD CARE implementation, • Twelve interviews were conducted — one per VAMC • One VAMC did not participate in the semi-structured interview • Number of clinicians per interview ranged from 1 to 4 • Interviews lasted 60–75 min and were conducted over Microsoft Teams^TM^ by trained VA pharmacy intern • Interviews were recorded, auto-transcribed, and reviewed for accuracy	Interviews conducted 8–12 months after completing the 5-week Academy

### Measures

Descriptions and data sources for each RE-AIM domain are detailed in Table 2.

**Table 2:** Description and data source for RE-AIM domain measures

RE-AIM domain	Domain operationalization	Data source(s)
**Reach**	• Number/type of clinicians who participated in the Academy • Number of front-line clinicians who were trained after the Academy	• Administrative records of: o Implementation team members and, o Front-line clinician training completion
**Effectiveness**	• Academy impact on clinicians’ self-efficacy or perceived capability in accomplishing implementation tasks [24].	• Survey items assessing perceived capability completing implementation tasks before and after Academy participation. o Scale of 1 = “not at all capable,” 5 = “moderately capable,” and 10 = “highly capable”
**Adoption**	• Proportion of clinicians that attended Academy sessions and used Academy resources	• Administrative program records of Academy completion, • Survey items assessing session attendance and use of resources. • Semi-structured interview informed understanding of participants’ reasons for participating in the Academy
**Implementation**^**a**^	• Clinicians’ perceptions of the acceptability and appropriateness of the Academy as an implementation package *• Acceptability* conceptualized as satisfaction with the Academy content and delivery approach *• Appropriateness* of the Academy = clinician’s perceived fit, usefulness, and practicality of the Academy with their VAMC [25].	• Survey items assessing Academy content and delivery approach, and relevance and usefulness of the Academy. • Semi-structured interviews offered specific examples of what clinicians valued about the Academy and ways it facilitated or hindered implementation
**Maintenance**	• Clinicians continued use of Academy tools, resources, and information provided several months after participation	• Semi-structured interview items about ongoing use of Academy tools and resources and plans for the next 6 months

^a^The *implementation* dimension is different than the RE-AIM conceptualization and instead draws on Proctor Implementation Outcomes of *acceptability* and *appropriateness*

### Data analysis

Descriptive statistics were calculated for all quantitative survey items and thematic analysis was used to summarize the qualitative open-ended items. To assess changes in self-reported capability, the non-parametric Wilcoxon signed-rank test was used for the 10 Likert scale items. No adjustments for repeated testing were made and an alpha level of 0.05 was used. IBM SPSS Statistics (Version 28) [27] was used for the statistical analysis. For structured interview data*,* descriptive statistics were calculated to summarize the eight Likert scale items and frequencies were calculated for the dichotomous yes/no items. An independent evaluator with no direct affiliation to the VA conducted a thematic analysis of the open-ended items separately, and a consensus of final themes was agreed on through group discussion with the project lead. The thematic analysis was conducted using NVivo [28]. Initially, an inductive approach was taken using open coding. Survey and interview findings are presented within the RE-AIM framework to identify indicators of the RE-AIM effectiveness and implementation outcomes for each domain. We used the SQUIRE 2.0 reporting guidelines when writing this paper [29].

## Results

The results are presented for each RE-AIM domain.

### Reach

Thirty-six clinicians from 13 VAMCs participated in the Academy in Fall 2020 and Spring 2021. Across all 13 sites, 264 front-line clinicians completed the COPD CARE clinician training. This group included 130 pharmacists, 117 nurses, and 17 other front-line clinicians (e.g., *respiratory therapists).*

### Effectiveness

Sixteen clinicians who served as clinician leads and implementation leads from the 13 VAMCs responded to the survey. Thirteen (81%) respondents were Clinical Pharmacist Practitioners and three (19%) reported other professions (i.e., inpatient care med-surg Tele Nurse). Clinicians reported significant increases in their capability to complete implementation efforts after participation in the Academy across ten items representing implementation tasks (*p* < 0.05) (Table 3).

**Table 3:** Changes in clinician capability to complete implementation tasks before and after Academy participation

Implementation task	Median range			
	Before Academy	After Academy	*Z*	*p*-value
Capability to gain support from leadership to initiate an interprofessional COPD CARE transitions program	5.0	8.5	2.953	0.003
Capability to coordinate the use of CPRS templates for COPD management	5.0	8.0	2.680	0.007
Capability to launch a COPD clinical training program	4.5	9.0	3.305	< 0.001
Capability to design a care transition patient referral process for COPD management	4.5	8.0	3.423	< 0.001
Capability to provide continued clinical updates for COPD management	5.0	8.0	3.192	0.001
Capability to form collaborations with services for COPD referrals	5.0	8.0	3.190	0.001
Capability to implement materials (e.g., COPD action plan) in clinic for COPD management	5.0	9.0	3.310	0.001
Capability to embed your profession within the COPD management team	5.0	8.0	3.078	0.002
Capability to launch the COPD CARE service at your facility	4.0	9.0	3.533	< 0.001
Capability to positively impact the lives of Veterans with COPD	5.0	10.0	3.195	0.001

*COPD* chronic obstructive pulmonary disease, *COPD CARE* Chronic Obstructive Pulmonary Disease Coordinated Access to Reduce Exacerbations, *CPRS* Computerized Patient Record System

### Adoption

Over 90% of clinicians responding to the survey reported complete or nearly complete attendance at all five of the Academy weekly discussions. Clinicians from 12 (92%) VAMCs participated in the semi-structured interview; one VAMC declined the request for an interview. Interviewed clinicians reported high utilization of Academy resources, with the workbooks being used by clinicians at all 12 (100%) VAMCs, followed by 11 (92%) VAMCs using the live virtual debrief meetings and the COPD CARE resources available through a shared network drive. Interview responses indicated that clinicians at three-fourths or more of VAMCs reported using the Academy weekly emails (83%), weekly YouTube videos (75%), and monthly post-Academy follow-up meetings (75%).

### Implementation

#### Acceptability

Clinicians’ perceptions of the Academy content and delivery approach suggest they were satisfied with these aspects of the Academy [30]. Interview findings indicate that clinicians at all 12 (100%) sites viewed the Academy content to be complete and covering critical aspects of implementing the COPD CARE service and clinicians at 83% of VAMCs reported the approach to delivering Academy content was effective.

Clinicians reported that they valued the team-based support aspect of the Academy including the opportunities to be part of the virtual discussions and a learning collaborative. Survey results indicated that nearly all clinicians (94%) found that learning from colleagues at other VAMCs during the Academy and attending the weekly live sessions (81%), were some of the most valuable aspects of the Academy.

The interviews corroborated the survey findings. Table 4 presents representative clinician quotes. Related to the Academy clinical training support, clinicians at about 75% of VAMCs viewed the clinical training content as helpful for preparing front-line clinicians to deliver COPD CARE. This was reiterated in the interviews with a clinician sharing that the clinical training content had far-reaching beneficial effects on increasing clinician comfort and motivation to treat COPD (Table 4). Clinicians shared how valuable it was to have the opportunity to problem-solve with clinicians from other VAMCs. However, clinicians also reported the Academy lacked sufficient content in certain areas (e.g., exploring spirometry in greater depth, additional resources to describe the COPD CARE referral process, and additional informatics support) (Table 4).

**Table 4:** Themes and representative quotes

Theme	Quotes
**RE-AIM domain — implementation**	
***Acceptability of COPD CARE Academy***	
Opportunity to problem-solve with other clinicians	I really find the work groups, very invaluable to talk with other sites that are implementing to share ideas, there’s been a handful of folks from my implementation group that I actually had…one on one meetings with either because they had challenges or I had challenges.…So, I really feel like the networking and kind of the using those other person resources, has been one of the most helpful aspects of it [Academy]. (CL10)
Clinical-training support	But I did think, …it was really helpful to have the clinical layout for how to teach it [COPD CARE Clinician training]. Because sometimes it’s hard to lay that out linearly from my own head. So,…I thought it was really helpful to have, like, the nursing training handbook and the pharmacist training handbook and have those things set out for you so that you can use it as a jump off point to teach. (CL18)
Clinician training increased comfort in treating COPD	I feel like for us the actual training modules for…the pharmacist…was the most helpful because…COPD isn’t something that we’ve done with Med management ourselves at all. So it’s not something we had a lot of comfort with to begin with, so I think getting a lot of that background information was really good for us because I mean, we’re used to like doing the hypertension and the diabetes and things, and that’s kind of our comfort zone, so getting more information to go past that I think was the most helpful. (IL13)
Academy lacked sufficient clinical content	[…] I think it would have been helpful to have like an subject matter expert…like a pulmonologist, kind of walk us through…the PFTs [pulmonary function tests] like I still find it challenging to read some of the PFTs you know as a pharmacist, you know I know about the drugs, but reading the PFTs may be challenging…So, that…would be… great if we had, you know, a session. (CL1)
Academy lacked sufficient guidance and resources for referral process	I know for us like one of our hiccups…is the Cadillac versus the Ford Model [COPD CARE referral model], and I felt like…I didn’t maybe have the best resources in doing it [setting up the referral model] ...That was maybe like a gap. So I don’t know if that…was lacking a little in that session or if there could have been more, maybe expanded on it or if we could have had a different session and invited the people that may be doing that [using the COPD CARE Referral tool]. But I just think…there could be room for improvement in those resources. (IL14)
***Appropriateness of COPD CARE Academy***	
Step-by-step approach was appropriate	There was a lot of infrastructure built into the [Academy] workbook, in a stepwise fashion. The steps are well thought out and made sense you know getting leadership support, making sure that we had the supplies on hand…I am an experienced supervisor, I’ve built a bunch of clinics before so the documentation [Academy workbook] of how to…build the clinics…was spot on….Especially if I was a new supervisor or…a frontline staff pharmacist, I would have a good idea of how to communicate that build. (IL3)
Facilitated accountability	[…] the accountability of having…the weekly [Academy] sessions. …that accountability of hey, we should be moving along with this, it’s easy whenever you are just given a workbook and said, okay implement this to just be like, oh, I’ll do it later, but when you have to check in each week and, say what your progress, is it puts a little bit more pressure to get it done right. (IL7)
External implementation approach was motivating	[…] [National COPD CARE facilitator] has been really…supportive and encouraging…he [National COPD CARE facilitator] said…it doesn’t have to be perfect. Starting off, as long as you…do something and start something, and then grow it as you’re able to, I think that was very encouraging and I think it motivated me … to just…start something within our health system, even though it may not be as robust or as… interdisciplinary as other sites. (IL2)
Academy topics not aligned with VAMC implementation phase	[…] the COPD CARE Academy was very prescriptive…week one to week six. Well, I may still have been working on tasks from week 2. But, you know, sometimes getting the right engagement and service involvement from these other folks, took several weeks to do…So…as we got to the later weeks of COPD CARE [Academy] where other sites were implementing kind of adjacent to the timing of the weekly schedule, our timeline was very, very different and so I think…it [Academy] became less and less applicable because it was on like Step six, and I’m still trying to get step two moving. (IL10)
Academy content and informatics tools are pharmacist centric	I think a lot of the focus [of COPD CARE Academy] as far as like the education goes was very pharmacist-centric…There wasn’t so much about how the nurse is actually involved in the process and what they’re doing for follow-up…and the pharmacist note template, it has a lot of detail in it …but the nursing template seemed a little bit sparse…I don’t think they [Nurses] had as much feel for what they should be doing as part of the process as well. (IL13)
**RE-AIM domain — maintenance**	
Continued COPD team communication after Academy completion	I attended…the COPD or the gold conference. I did the virtual, so they have lots of good presentations and so each week since we started the COPD CARE launch in January, I’ve been sending weekly emails [to clinicians implementing COPD CARE at site] and just asking for questions and then trying to follow up with questions and things that we’re noticing. (IL7)
Integration of Academy training into existing meetings	[…] we have a twice a year, education day and so our fall one was…I think it was half the day that was dedicated to COPD CARE so we all sat through the modules to get there and watch things together... (IL13)
Long-term use of Academy resources	[…] I think that the handout provided and all the videos were really well done. I think most of us here we’re kind of unfamiliar with COPD. And then, and then after going through it, again, I think the training was just was really good and the handouts, we still use we reference now has been a couple of months… (CL14)
Benefit of monthly post-Academy meetings	I mean some of the information that’s been ongoing discussion in the community working group has been more helpful, because a lot of the questions my clinical pharmacists have were just like those higher level nuanced you know specific case questions like, some of the things we’ve been talking about as far as like steroids deprescribing or concomitant asthma diagnosis. (IL9)

*CL* clinician lead, *IL* implementation lead

#### Appropriateness

Clinicians at nearly 70% of VAMCs perceived the Academy to be critically important and clinicians at 75% of VAMCs reported having the necessary tools and resources to implement COPD CARE after Academy participation. Clinicians found the external IF approach to be appropriate. Participants felt supported, motivated, and encouraged by the support from the national facilitator (Table 4). However, some clinicians reported challenges to Academy participation; describing ways in which the Academy may not have been perceived as suitable or practical as an implementation package for those VAMCs. For example, one clinician found that the weekly Academy topics were not always aligned with where their VAMC was in the implementation process (Table 4).

### Maintenance

Clinicians from 92% of responding VAMCs reported long-term utilization of Academy resources and clinicians from 75% of VAMCs reported participating in the post-Academy meetings with other participants. The Academy had a lasting effect on sites, which was reflected in VAMC’s integration of the Academy into the site organizational structure. Several clinicians reported that their implementation teams continued to have regular communication after the Academy (Table 4). For many, the Academy served as a lasting resource. Clinicians reported long-term use of the training materials and resources months after the Academy, suggesting its lasting effect and value (Table 4).

## Discussion

Guided by the RE-AIM framework we evaluated the Academy’s impact on building capacity for implementing COPD CARE, including clinicians’ perceived capability to implement COPD CARE. The use of IF as the overarching approach paired with additional strategies seemed to demonstrate positive outcomes across all RE-AIM domains. In this evaluation, we found that the fully-virtual, cohort-based IF approach was successful at building capacity for implementing COPD CARE at a large number of VAMCs simultaneously. This approach eliminates geographic and cost barriers to participation, increasing the reach of the Academy.

Interview findings suggested clinicians were committed to Academy participation and had a high degree of resource utilization, indicating successful adoption. A majority of clinicians were satisfied with the content and delivery approach and viewed the Academy as a useful and practical approach to implementing COPD CARE, which are indicative of successful implementation. These positive RE-AIM implementation outcomes likely contributed to the effectiveness of the Academy at increasing clinicians’ perceptions of their capability to implement COPD CARE. As clinicians embraced the Academy and participated in the virtual discussions with other VAMCs, they felt supported and their perceived capability to be successful at implementing COPD CARE increased. The linkage between the Academy’s strategies and increasing perceived capability or self-efficacy is supported by Bandura’s Social Cognitive Theory [31].

Findings from this evaluation add to the literature on the development of internal facilitators to build capacity for implementing best practices [23, 32, 33]. The Academy participants reported receiving the guidance and resources they needed to successfully implement, suggesting that the external facilitators provided the necessary knowledge and guidance for skill development [32]. The Academy supported relationship building and the creation of a supportive environment, which was reflected in clinicians’ reporting that they valued the opportunity to interact and problem solve with the external facilitators as well as with other internal facilitators from other VAMCs who were implementing simultaneously. The Academy also provided training in COPD management and materials and support for clinicians to train their colleagues, which is a necessary support for internal facilitators in healthcare settings [33].

Our findings suggest that the Academy was successful in enhancing clinician’s perceived capability to complete implementation tasks, which likely contributed to minimizing barriers to implementing COPD best practices [9, 10]. To promote practitioner engagement and address limited staffing, the Academy provided training and resources for clinicians to engage with colleagues in other disciplines (e.g., nursing, respiratory therapy) and obtain their buy-in to collaborate on the implementation of COPD CARE. This is reflected in our finding that clinicians’ reported increased capability to gain support from leadership for COPD CARE and to form collaborations with other services after participation in the Academy. The development of such collaborations promotes sharing of staffing responsibilities, addressing barriers related to limited staffing. The Academy also promoted the development of an informatics infrastructure through the provision of COPD CARE referral dashboards and clinical note templates. Clinicians were guided through the process of initially installing informatics tools and were provided training materials to support them in training their clinician colleagues in the use and application of the informatics tools.

Despite the positive outcomes in our evaluation, some clinicians identified challenges to participating in the Academy and highlighted content or resources they perceived to be lacking, such as additional training and informatics support to improve patient referrals. Opportunities exist to explore this barrier and enhance the COPD CARE referral process.

There were some potential limitations to this evaluation. First, clinicians assessed their pre-Academy capabilities after Academy participation, which introduces the potential for bias. This was mitigated in part by discussing their perceptions of the Academy on their capabilities during the interview. Second, some of the interviews involved multiple clinicians from a VAMC, which may have affected their willingness to openly share their experiences with the Academy. To the extent possible, interviewers made efforts to create a safe environment to facilitate honest feedback. Third, some clinicians did not participate in the interviews and important differences in their viewpoints may not have been captured in data collection. While there was variation in interview participants across VAMCs, the evaluation team ensured that at least one clinician who was designated as an implementation lead was involved in each interview. Future evaluations will more carefully consider sampling approaches. A limitation of this evaluation is its sole focus on the front-line clinician perspective and not other stakeholders’ (e.g., leadership, clinic managers) perspectives. Furthermore, VAMCs engaged in this evaluation elected to implement COPD CARE, which may have increased clinician motivation to complete the Academy. Notably, these efforts to disseminate COPD CARE were made during a global pandemic, clinicians were not provided with additional salary support or protected time for their implementation efforts, and national facilitation of the program relied heavily on part-time VA pharmacy interns.

## Conclusions

Through this evaluation, we documented the impact the Academy had on enhancements to clinician perceptions of capability to implement successfully. We also identified potential areas of improvement for the Academy as an implementation package to support the scale-up of COPD CARE. These lessons learned are important to inform future Academy improvements as it is rolled out to additional VAMCs. This evaluation adds to the growing evidence base supporting the efforts to scale COPD CARE. It builds on a previous iteration of the implementation package focused solely on clinician training and paves the way for future evaluations to further examine the Academy’s impact and future iterations of post-Academy strategies.

## Data Availability

The datasets used and/or analyzed during the current study are available from the corresponding author on reasonable request.

## Abbreviations

COPD: Chronic obstructive pulmonary disease
COPD CARE: Chronic Obstructive Pulmonary Disease Coordinated Access to Reduce Exacerbations
IF: Implementation facilitation
PFTs: Pulmonary function tests
VA: Veterans’ Health Administration
VAMC: Veterans’ Health Administration Medical Center
